# Supplementary material for: The Combination of Oolonghomobisflavan B and Diallyl Disulfide Induces Apoptotic Cell Death via 67-kDa Laminin Receptor/Cyclic Guanosine Monophosphate in Acute Myeloid Leukemia Cells
Source: Curr Issues Mol Biol. 2024 Mar 14;46(3):2444–55. doi: 10.3390/cimb46030154 (PMC10969046; doi:10.3390/cimb46030154)
Supplement: Supplementary file 1 [file cimb-46-00154-s001.zip › cimb-2894683-supplementary.pptx]

## Slide 1
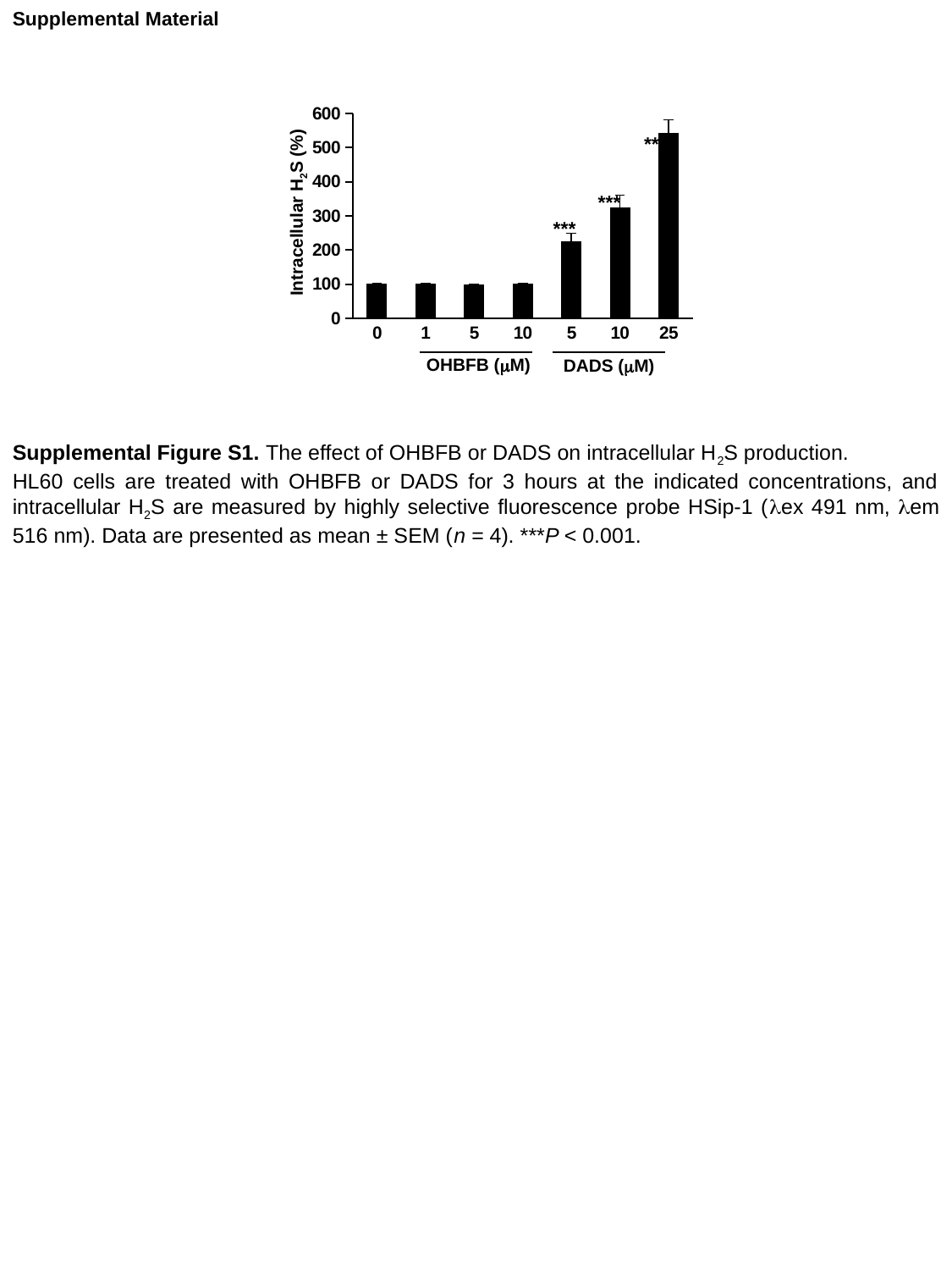

Supplemental Material
### Chart
| Category | |
|---|---|
| 0.0 | 100.0 |
| 1.0 | 100.3829981960951 |
| 5.0 | 97.72164892237313 |
| 10.0 | 99.84587960997924 |
| 5.0 | 223.4437635587625 |
| 10.0 | 324.2263031149782 |
| 25.0 | 541.8345668859646 |***
***
 Intracellular H2S (%)
***
OHBFB (mM)
DADS (mM)
Supplemental Figure S1. The effect of OHBFB or DADS on intracellular H2S production.
HL60 cells are treated with OHBFB or DADS for 3 hours at the indicated concentrations, and intracellular H2S are measured by highly selective fluorescence probe HSip-1 (lex 491 nm, lem 516 nm). Data are presented as mean ± SEM (n = 4). ***P < 0.001.

## Slide 2
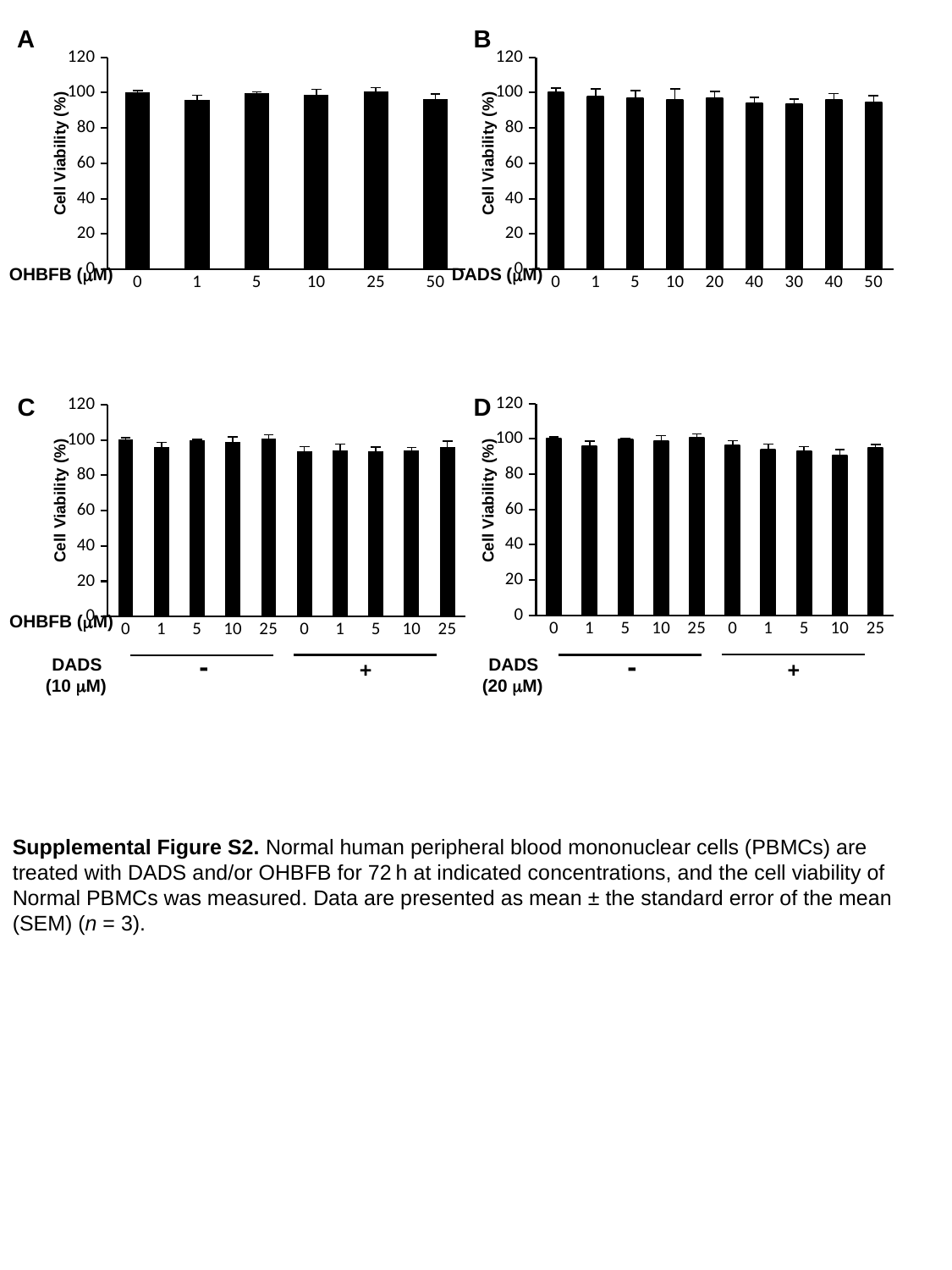

A
B
### Chart
| Category | |
|---|---|
| 0.0 | 100.0 |
| 1.0 | 95.66666666666667 |
| 5.0 | 99.66666666666667 |
| 10.0 | 98.66666666666667 |
| 25.0 | 100.6666666666667 |
| 50.0 | 96.33333333333331 |
### Chart
| Category | |
|---|---|
| 0.0 | 100.0 |
| 1.0 | 98.0 |
| 5.0 | 97.0 |
| 10.0 | 96.0 |
| 20.0 | 97.0 |
| 40.0 | 94.0 |
| 30.0 | 93.66666666666667 |
| 40.0 | 96.0 |
| 50.0 | 94.33333333333331 | Cell Viability (%)
 Cell Viability (%)
OHBFB (mM)
DADS (mM)
C
D
### Chart
| Category | |
|---|---|
| 0.0 | 100.0 |
| 1.0 | 95.66666666666667 |
| 5.0 | 99.66666666666667 |
| 10.0 | 98.66666666666667 |
| 25.0 | 100.6666666666667 |
| 0.0 | 96.33333333333331 |
| 1.0 | 94.0 |
| 5.0 | 93.0 |
| 10.0 | 90.66666666666667 |
| 25.0 | 94.66666666666667 |
### Chart
| Category | |
|---|---|
| 0.0 | 100.0 |
| 1.0 | 95.66666666666667 |
| 5.0 | 99.66666666666667 |
| 10.0 | 98.66666666666667 |
| 25.0 | 100.6666666666667 |
| 0.0 | 93.33333333333331 |
| 1.0 | 94.0 |
| 5.0 | 93.33333333333331 |
| 10.0 | 93.66666666666667 |
| 25.0 | 95.66666666666667 | Cell Viability (%)
 Cell Viability (%)
OHBFB (mM)
-
+
-
+
DADS
(10 mM)
DADS
(20 mM)
Supplemental Figure S2. Normal human peripheral blood mononuclear cells (PBMCs) are treated with DADS and/or OHBFB for 72 h at indicated concentrations, and the cell viability of Normal PBMCs was measured. Data are presented as mean ± the standard error of the mean (SEM) (n = 3).
